# Patterns of Gene Flow Define Species of Thermophilic Archaea

**DOI:** 10.1371/journal.pbio.1001265

**Published:** 2012-02-21

**Authors:** Hinsby Cadillo-Quiroz, Xavier Didelot, Nicole L. Held, Alfa Herrera, Aaron Darling, Michael L. Reno, David J. Krause, Rachel J. Whitaker

**Affiliations:** 1 Department of Microbiology and Institute for Genomic Biology, University of Illinois, Urbana-Champaign, Urbana, Illinois, United States of America; 2 Department of Statistics, University of Oxford, Oxford, United Kingdom; 3 Genome Center, University of California, Davis, Davis, California, United States of America; University of Edinburgh, United Kingdom

## Abstract

Despite a growing appreciation of their vast diversity in nature, mechanisms of speciation are poorly understood in *Bacteria* and *Archaea*. Here we use high-throughput genome sequencing to identify ongoing speciation in the thermoacidophilic Archaeon *Sulfolobus islandicus*. Patterns of homologous gene flow among genomes of 12 strains from a single hot spring in Kamchatka, Russia, demonstrate higher levels of gene flow within than between two persistent, coexisting groups, demonstrating that these microorganisms fit the biological species concept. Furthermore, rates of gene flow between two species are decreasing over time in a manner consistent with incipient speciation. Unlike other microorganisms investigated, we do not observe a relationship between genetic divergence and frequency of recombination along a chromosome, or other physical mechanisms that would reduce gene flow between lineages. Each species has its own genetic island encoding unique physiological functions and a unique growth phenotype that may be indicative of ecological specialization. Genetic differentiation between these coexisting groups occurs in large genomic “continents,” indicating the topology of genomic divergence during speciation is not uniform and is not associated with a single locus under strong diversifying selection. These data support a model where species do not require physical barriers to gene flow but are maintained by ecological differentiation.

## Abstract 2

A genomic view of speciation in Archaea shows higher rates of gene flow within coexisting microbial species than between them.

## Author Summary

Microorganisms from the bacterial and archaeal domains of the tree of life comprise the greatest breadth of biodiversity on earth. Yet the essential evolutionary process of speciation (through which biodiversity is generated) is poorly understood in microbes. At issue is the fundamental question of whether gene flow among individuals of clonally reproducing microorganisms is rapid enough to provide coherence within—and prevent speciation between—coexisting lineages. We use complete sequencing of microbial genomes to observe speciation in action. We focus on *Archaea* called *Sulfolobus islandicus* gathered from a geothermal hot spring from the Mutnovsky Volcano in Kamchatka, Russia, whose physical isolation allows us to pinpoint evolutionary processes to one location. Contrary to the theoretical predictions for microbes, we provide evidence that two novel lineages are in the process of becoming ecologically distinct and evolutionarily independent despite the fact that they recombine. The divergence we observe is not happening uniformly across the genome because certain genomic regions are more prone to become differentiated between species than others. This genomic view of the process of speciation occurring within a single natural microbial population contributes to our understanding of the generation of biodiversity in *Archaea* and furthers our understanding of speciation across the tree of life.

## Introduction

Molecular sequence analyses of microbial populations commonly reveal discrete clusters of sequence diversity indicative of closely related, but distinct, coexisting, lineages [1]–[9]. Such clusters are sometimes given the status of species, especially when they are shown to be ecologically distinct [6]. Resolving the evolutionary mechanisms that cause the formation and maintenance of these clusters holds the key to understanding the process of speciation in clonally reproducing, asexual microorganisms [10].

In the absence of geographic barriers, speciation depends upon the balance between gene flow holding lineages together and selection pulling them apart [11],[12]. For asexual microorganisms, two primary theoretical models explain the formation of sequence clusters, each tipping the scale in the opposite direction of the recombination-selection balance. The first emphasizes the importance of selection driving ecological specialization. This model predicts that the persistent coexistence of sequence clusters can result from sequential selective sweeps of adaptive mutations to different niches [13]. Persistent “ecotypes” are held together by the cohesive force of genetic drift or periodic selection and kept apart by selection for niche-specific adaptations or against niche-specific maladaptations [13],[14]. This model can incorporate low levels of gene flow of universally adaptive genetic material without limiting the effects of periodic selection differentially occurring in the ecotypes [15]. Ecological differentiation, correlated with decreased recombination, has been observed using multilocus sequence markers [6] and recently through whole genome analysis in *Escherichia coli* strains that have evolved to inhabit different environments [16].

A second model relies on barriers to recombination in the absence of selection to explain persistent sequence clusters [17]. It demonstrates that clusters will form if the effects of recombination are lower than mutation. In this neutral model, when recombination is greater than mutation, recombination plays a cohesive role that is strong enough to prevent the formation of persistent independent clusters of sequences, unless there is a significant physical barrier to gene flow between them [17]–[19]. For microorganisms, many such barriers can be hypothesized [20]. The most often mentioned and broadly distributed among microbial taxa is caused by mismatch repair recognition [21], which reduces the frequency of homologous recombination (HR) between divergent sequences. This type of barrier has been shown to lead to the formation of persistent diverging and independent clusters of sequences by allowing recombination within but not between groups that diverge through genetic drift [17],[22].

Whether recombination barriers or selection play the primary role in driving divergence of sequence clusters and whether the balance between these processes results in the maintenance of independent species in microorganisms is a controversial topic [16],[23],[24]. Many *Bacteria* and *Archaea* exhibit significant rates of HR and other forms of horizontal gene flow [25]–[28]. However, for each of the known mechanisms of horizontal transfer (transduction, transformation, and conjugation) only a small region of the chromosome may be transferred with each event. Whether this level of recombination among coexisting strains is strong enough to overcome ecological specialization and periodic selection and how the balance between recombination and selection will affect the topology of speciation across the chromosome in microorganisms is only beginning to emerge with the advent of whole genome sequencing [16],[29].

To examine the mechanisms of divergence and maintenance of independent species in *Archaea*, we sequenced the complete genomes of 12 strains of the thermoacidophilic Archaeon *Sulfolobus islandicus* from a single hot spring from the Mutnovsky Volcano region in Kamchatka, Russia. This location was selected because the *S. islandicus* population from the Mutnovsky volcano has been shown to be geographically isolated [30],[31], thus allowing us to investigate evolutionary processes occurring within well-defined geographic boundaries.

## Results and Discussion

**Figure 1:**
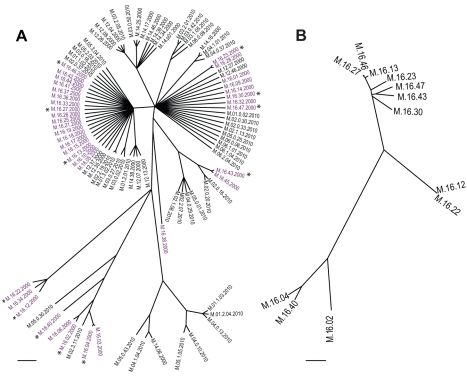
Phylogenetic relationships among *S. islandicus* from the Mutnovsky Volcano. (A) ClonalFrame [35] reconstruction based on seven loci from 97 *S. islandicus* strains from the Mutnovsky Volcano region of Kamchatka, Russia (details in Table S1). Strains in purple were isolated from spring M.16. The first number in each name indicates the spring from which strains were isolated, the second indicates the isolate number from that spring, and the third indicates year of isolation. * designates strains selected for genome sequencing and comparison. (B) ClonalFrame phylogeny based on the core genome alignment of 12 *S. islandicus* strains from hot spring M.16.

Multilocus sequence analysis (MLSA) using a set of seven loci [32] from 97 *S. islandicus* strains from nine hot springs sampled from the Mutnovsky Volcano region in the years 2000 and 2010 (listed in Table S1 and Table S2) showed significant differentiation by F_ST_ [33],[34] among springs in only a few cases (Table S3) [25]. No differentiation was observed when strains were pooled by year. The M.16 spring was chosen for further analysis because the diversity within this spring represents the diversity of the Mutnovsky population as a whole (Figure 1A). To further investigate patterns of gene flow within this population, the genome sequences of 10 new *S. islandicus* M.16 strains all from a single sample collected in 2000 were compared to two strains that have been previously sequenced from the same hot spring sample [30].

### Frequency of Homologous Recombination

The 12 *S. islandicus* genomes had an average size of 2.64 Mb (Table 1), of which approximately 86% was shared by all strains. The genomic sequences of the strains were very similar, with pairwise genetic distances ranging from 0.01% to 0.35% (Table 1). Shared genomic regions were used to infer the clonal genealogy using ClonalFrame, which accounts for the possibility of HR disrupting the signal of vertical genetic inheritance [35]. The relationships reconstructed using the full genomes (Figure 1B) confirmed those resolved by MLSA (Figure 1A), and further resolved bifurcations among strains that had unclear relationships using only seven marker loci. Based on this clonal genealogy, HR events were reconstructed using ClonalOrigin [36]. Overall, ClonalOrigin estimated that each nucleotide was substituted by recombination with a higher probability than mutation with a ratio estimated between 1.8 and 13 [37]. This value is above the threshold predicted to prevent divergence among sequence clusters in simulations of neutral populations with a small effective population size of 10^5^ [17].

**Table 1:** Characteristics of 12 strains of *S. islandicus* from spring M.16.

Group	Name	Size (Mb)	Pairwise Genetic Distance (%)			Growth Rate	Final OD
			M.16.27	M.16.4	M.16.12		
**Blue**	M.16.27^a^	2.69	—	0.32	0.19	0.005	0.25
	M.16.46	2.69^b^	0.00	0.32	0.19	0.003	0.13
	M.16.13	2.63^b^	0.01	0.32	0.19	ND	ND
	M.16.47	2.64	0.06	0.32	0.18	0.002	0.09
	M.16.23	2.6	0.05	0.31	0.18	0.003	0.17
	M.16.30	2.61^b^	0.06	0.31	0.17	0.003	0.14
	M.16.43	2.59	0.08	0.32	0.17	0.001	0.07
	M.16.12	2.69^b^	0.19	0.31	—	0.009	0.57
	M.16.22	2.68^b^	0.19	0.31	0.00	0.009	0.63
**Red**	M.16.02	2.65	0.35	0.18	0.35	0.007	0.45
	M.16.40	2.67	0.33	0.06	0.33	0.011	0.60
	M.16.4^a^	2.59	0.32	—	0.31	0.006	0.41

a Previously published in [30].

b Genome not closed.

ND, not done.

**Figure 2:**
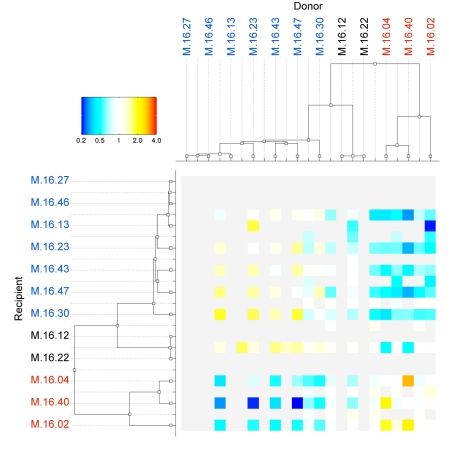
Heat map representation of homologous recombination frequency for every donor/recipient pair of branches of the core genome phylogeny of 12 *S. islandicus* strains. Recombination frequency is measured relative to its expectation under the prior of the ClonalOrigin model and color coded according to the upper left color/magnitude legend (light blue and blue for the frequency of recombination events below a 1∶1 ratio and yellow to red for the frequency of recombination events above 1∶1). Light gray cells represent non-significant ratios with less than four observed and expected events. White shows number of events that match the prior expectations. Names of strains are color coded as Blue and Red groups.

Using ClonalOrigin we were able to map recombinant fragments between donor and recipient genomes. As shown in Figure 2, the pattern of recombination between branches of the tree differed from the values expected under the coalescent model with constant recombination. The observed number of HR events was higher than expected within two groups, but lower than expected between them. The first group contains seven strains (M.16.27, M.16.46, M.16.13, M.16.23, M.16.43, M.16.47, and M.16.30), hereafter called the Blue group, and the second contains three strains (M.16.4, M.16.40, and M.16.02), hereafter called the Red group. This pattern of higher gene flow within than between two groups (Red and Blue) coexisting in the same hot spring fits the biological species concept [10],[38],[39]. The absolute numbers of events (Figure S1) show that there are rare transfers between divergent sets of strains in the Red and Blue groups, indicating that there is not a complete barrier to gene flow, but rather a relative decrease in the number of recombination events between groups.

The two intermediate and nearly identical strains (M.16.12 and M.16.22) receive recombinant fragments at a higher relative frequency from the Blue group than from the Red group (Figure 2) but serve as a donor at a lower frequency than expected to both the Blue and Red groups. We excluded them from further analysis because it was unclear whether they should be included in the Blue group or kept separate.

### Non-Homologous Gene Flow

Microorganisms such as *S. islandicus* engage in promiscuous, non-homologous gene flow through horizontal gene transfer, which is often mediated by integration of a diversity of mobile elements such as viruses and plasmids [30],[40]. To test whether there is evidence for non-homologous gene flow among populations, we mapped variation in genome content onto the core gene phylogeny. In total, we identified 48 non-core segments longer than 5 kb that are found in only some of the 12 strains (Table S4). Thirty-four of these can be explained by a single gain or loss event on a single branch along the phylogenetic tree (Figure 1B), including 12 gains by a single genome (Table S4). The remaining 14 non-core segments have distributions that require multiple events to be explained (e.g., gene flow between strains or gene gain followed by differential gene loss). Only four of these (36, 38–40 in Table S4) could result from exchange between strains in the Blue and Red groups. The distribution of non-core regions of the 12 *S. islandicus* genomes is therefore consistent with a low level of non-homologous gene flow between the two groups, analogous to the pattern of homologous gene flow observed above.

### Decreasing Gene Flow Between Groups

**Figure 3:**
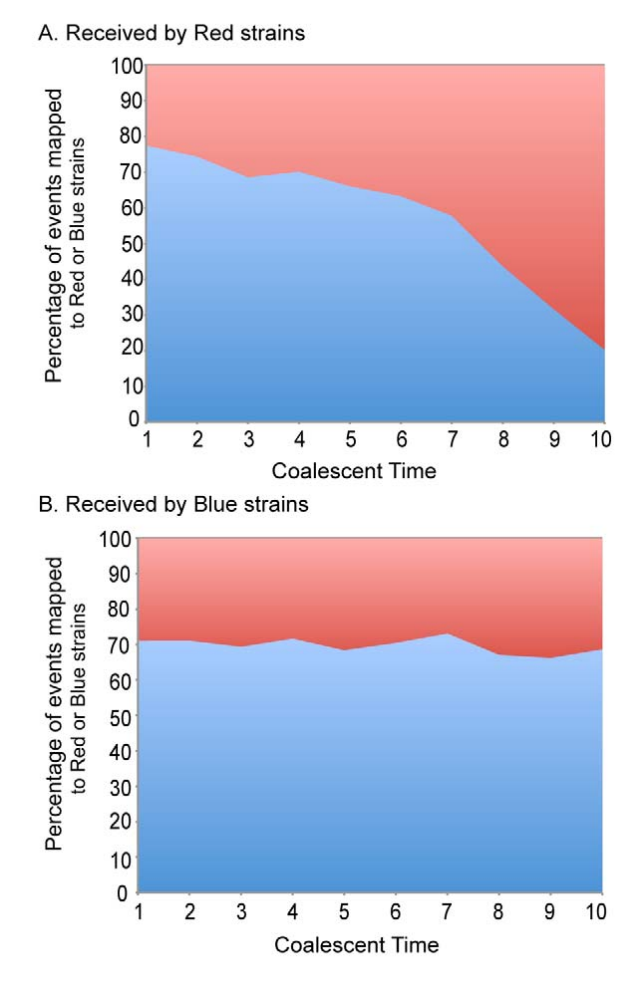
Variation in recombination events between the Red and Blue groups through time. For the Red (A) and Blue (B) recipient strains, the total proportion of events that could be assigned as originating from either donor Red (colored red) and Blue (colored blue) strains is shown as a function of coalescent time with 10 being the most recent divergence and 1 being the common ancestor of this set of strains. A coalescent unit of time is equal to the average length of a generation multiplied by the effective population size.

The Red and Blue groups identified in Figure 2 could be either diverging over time or could have evolved independently and started to converge through a process of multiple migration with introgression [41],[42]. To differentiate these two hypotheses, we studied the distribution of recombination events between the two species through coalescent time as inferred by ClonalOrigin. Figure 3 shows that the percentage of the recombinant events from the Blue to the Red groups is decreasing over coalescent time (Figure 3A). This indicates that the groups are progressively diverging in a manner that is consistent with ongoing speciation.

As shown by MLSA in Figure 1A and Table S3, high levels of differentiation do not occur between hot springs. This indicates that the speciation we have observed in one spring has in fact occurred at a larger scale within the Mutnovsky population, which has been shown to be geographically isolated when compared to similar populations from North America [30],[31]. We cannot exclude the possibility that one of these two groups initially diverged elsewhere and migrated to the Mutnovsky Volcano. The decrease in gene flow between groups suggests that if this were the case, ongoing migration is decreasing over time. Our observation of recent, low levels of gene flow between groups demonstrates the coexistence and interaction of these groups of strains. Therefore, we investigated mechanisms that either drive or maintain the independence of these groups as they coexist.

### Absence of Physical Barriers to Gene Flow

Although recombination rates in this Archaeon, as in other microorganisms, are low relative to sexual eukaryotes, the two primary models for speciation in microorganisms predict that either barriers to recombination, or diversifying selection, between the two types is necessary to explain their maintenance and ongoing divergence. We first investigated possible physical barriers to recombination between the two species. Neutral divergence of lineages can occur when there is a decrease in recombination with genetic divergence resulting from mismatch recognition [21]. This relationship has been identified in many bacterial and eukaryotic species [43],[44]. We tested this hypothesis by examining whether regions of the chromosome with higher divergence exhibited lower frequencies of recombination than those with lower divergence. This analysis therefore looks at the variation in rates of recombination along the chromosome rather than between particular partners as above. We found no correlation between genetic divergence and recombination frequency when all recombination events from the populations were pooled (slope 0.016, *R* ^2^ = 6.3e-07, Figure S2A). This contrasts with (i) the same analysis in the *Bacillus cereus* group [36], where a negative correlation was found (slope −3.28, *R* ^2^ = 0.29, Figure S2B), (ii) experimental data for *Bacteria* tested over the same range of genetic distances (Figure S2C) [17],[43]–[46], and (iii) metagenomic analysis of the Archaeon *Ferroplasma* [47]. This lack of correlation between sequence divergence and rate of recombination is, however, consistent with the mechanisms of recombination reported for another *Sulfolobus* species, *S. acidocaldarius*, in which short tracts of 20–22 nt are incorporated during transformation with sequence identity of only 2–3 nt required at either end of the import [48]. In addition, the lack of an identified *mutSL* system in this species may result in the failure to prevent recombination between divergent sequences [49].

Homologous recombination and non-homologous gene flow through mobile elements has been observed in laboratory cultures between strains of *S. acidocaldarius* [50],[51]. In this system, DNA transfer between cells is thought to occur through pilin-mediated aggregation and conjugation [51],[52]. We considered physical barriers that could prevent aggregation and conjugation among sympatric species of *S. islandicus*. The most highly variable sequence in the genome is that of the large subunit of the S-layer protein that covers the cell surface of *S. islandicus* and other microorganisms [53]. With one exception, alleles of the S-layer cluster into three distinct groups: Blue, Red, and the strains M.16.12 and M.16.22. The notable exception, M.16.30, is the most divergent Blue strain, which possesses an allele most similar to the Red group (Figure S3). The incongruence of the M.16.30 strain possessing the Red allele but showing a history of recombination with the Blue strains suggests that S-layer divergence did not pose the barrier to recombination that resulted in the divergence of the Red and Blue groups.

The Red and Blue S-layer alleles are highly divergent (with 12% nucleotide substitutions) compared to the rest of the genome, especially in the surface regions of the protein that are shown to be highly glycosylated in other species [54]. The allele in the Blue group appears to have been acquired by horizontal gene transfer, as it does not fit the core gene phylogeny of other sequenced *Sulfolobus* strains (Figure S3) [30]. Two amino acid changes are identified between the Red and Blue alleles of the *ups* pili believed to be responsible for aggregation and possible DNA exchange in contact with the S-layer [52],[55]. We cannot exclude the possibility that the history of recombination shown in M.16.30 is not consistent with its current genotype because of the recent acquisition of the novel allele by the Blue group or the recent transfer of this allele between a strain belonging to the Red group and M.16.30. In this case, the S-layer may serve as a barrier to current gene transfer, analogous to the prezygotic barrier in macroorganisms, possibly reinforcing the isolation between lineages following their initial differentiation.

The specificity of restriction enzymes could serve as barrier to recombination between species [20]. Because restriction enzymes are difficult to recognize bioinformatically, we investigated the distribution of methyltransferases that can confer specific protection to their linked restriction systems among the genomes of our 12 *S. islandicus* strains. While variation exists between strains, none of it was consistent with the distinction between the Blue and Red groups of *S. islandicus* strains, indicating that these systems are not providing a barrier to genetic exchange between the two groups. Although there may be physical barriers to the transfer and integration of DNA between the Red and Blue groups of strains that we have not yet identified, the set of possible mechanisms we have tested thus far do not appear to contribute to the decrease in gene flow between the Red and Blue groups that we observe.

### Genomic Signatures of Selection and Ecological Differentiation

In the absence of physical barriers to gene flow, diversifying selection may be driving these two incipient species apart or maintaining their differentiation. We note that the relatively low frequency of recombination observed in microorganisms, as compared with sexual eukaryotes, facilitates selection-driven divergence. We examined the differences in genotypes and phenotypes between the two groups to identify possible loci under differential selection that could cause ecological differentiation. First, we estimated the ratio of non-synonymous to synonymous rates of substitutions (d_N_/d_S_) between the Blue and Red groups and performed the MacDonald-Kreitman test [56] for all core genes. No indication of diversifying selection (values significantly greater than 1.0) was found. Overall, our estimate of the average d_N_/d_S_ was 0.42, which is consistent with recent divergence of these two groups in which purifying selection has not yet cleared mildly deleterious non-synonymous substitutions [57],[58].

**Figure 4:**
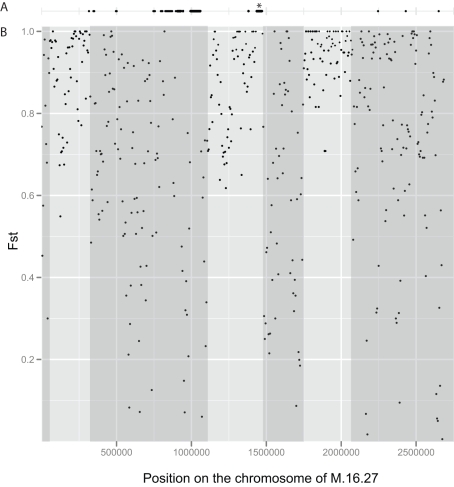
F_ST_ values between the Red and Blue groups along the chromosome of strain M.16.27. (A) 10,000 bp windows on the M.16.27 genome where genome sequence is not present in all 10 strains from the Red and Blue groups. These positions highlight variable portions of the M.16.27 genome. *Indicates a recently integrated plasmid. (B) F_ST_ values were calculated for sliding windows of 10 kb moving in 5 kb steps. Empty windows where sequence from M.16.27 is not shared by all strains are not plotted. Shading highlights regions of the chromosome that are less differentiated beginning and ending with the first window with F_ST_ values lower than 0.5.

Genomic loci that are under differential selection between two diverging species have been identified as outlier loci in genomic islands associated with divergent alleles differentially fixed between populations [59],[60]. In total, we identified 8,185 informative single nucleotide polymorphisms (SNPs) (excluding indels) within the Blue and Red groups of strains. Of these, 4,232 (52%) were fixed differences between the two groups. This high number of fixed differences between the two groups of strains relative to recently diverged sexual species [61],[62] may result from the relatively low rates of non-reciprocal gene flow occurring in *Sulfolobus*. We calculated F_ST_ values based on individual non-indel SNPs for each gene and in sliding windows of 10 kb across the genome (Figure 4). F_ST_ measures the level of differentiation between two groups [63],[64]. Low values indicate more diversity within than between groups that can result from either constrained divergence between groups by purifying selection or the exchange of alleles through recombination. High values indicate differentiation with more variation between groups than within. This occurs in regions that are under diversifying selection or where there are low levels of recombination [63]. This analysis revealed that the majority of the core chromosome exhibits high F_ST_ values and appears to be differentiated. Of the 1,883 genes shared between the Blue and Red groups in which variation was detected, only 466 genes (25%) exhibit F_ST_ values lower than 0.5. As shown in Figure 4, although the majority of the genome is highly differentiated (F_ST_>0.5) lower levels of differentiation (F_ST_<0.5) between the two species occur exclusively in three large regions of the genome (ranging in size from approximately 265 Kb to 770 Kb), separated by differentiated genomic “continents” (ranging in size from 290 Kb to 370 Kb) [65] where no lower values are observed. Within these larger regions, smaller differentiated islands, between 5 Kb and 230 Kb, in length exist separated by contiguous regions of low differentiation between 10 Kb and 30 Kb long.

The broad topology of differentiation across the chromosome is consistent with either differential selection among many loci within the chromosome (located within the three regions of high differentiation), different levels of gene flow in different regions of the chromosome (higher gene flow in regions of lower differentiation), or both [64],[65]. Many loci under weak selection would explain our failure to identify loci under diversifying selection using the patterns of non-synonymous to synonymous substitutions or the MacDonald-Kreitman test. This pattern is also consistent with the prediction that, due to the mechanisms of gene flow in microorganisms, different regions of the chromosome will exhibit different patterns of speciation [29]. Chromosomal regions that are less susceptible to recombination between species will differentiate first even if they are not associated with ecological differentiation. Interestingly, most non-core genes occur in regions of low differentiation between the two groups (Figure 4). This is inconsistent with novel genes driving differentiation between species or with the import of novel gene islands decreasing recombination and promoting speciation [16],[66]. This lack of differentiation in regions of the chromosome with variable gene content supports the earlier observation and experimental data that suggest long regions of high sequence homology is not required for recombination. While the basis for the existence of these genomic “continents” of differentiation is unknown, they highlight the importance of using whole genomes when investigating speciation.

To determine whether differences in gene content between the Red and Blue groups could be responsible for their ecological differentiation, we identified four contiguous regions of more than 5 Kb in which more than half of the genes are shared exclusively by all members of a group (Table S4). Two of these contain genes of unknown function (1 and 3, Table S4), with one having signatures of being an integrated plasmid fragment (1 on Table S4). Two additional islands, one in each species, with functional annotations, were identified. The first island (2 in Table S4), present only in the Red strains, contains six subunits (*Tmo*A αβ, B, C, D, and E) and three accessory proteins of a putative Toluene-4-monoxygenase system. This region is present in many previously sequenced strains of *S. islandicus* [30] and was probably lost by the Blue group after its divergence from the Red group. The Blue strains share an island of four subunits (α, β, δ, and γ) of a putative respiratory nitrate reductase system (4 in Table S4). Active nitrate reductases have been observed in other *Archaea* [67] but not in *Sulfolobales*, while a putative monooxygenase operon, similar to that in the Red group, was reported to be active in *S. solfataricus* [68]. In both of these regions, d_N_/d_S_ values and divergence were similar to the estimates for the rest of the genomes. Both of these islands occur in the large, second region of lower differentiation and high variation in gene content identified in Figure 4.

**Figure 5:**
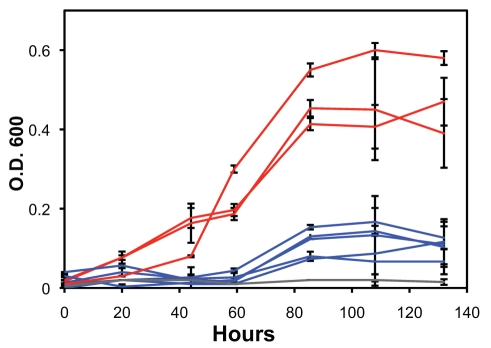
Growth of M.16 strains under standard heterotrophic conditions. Lines are color coded for strains assigned to the Red and Blue groups. Negative control with no inoculum added is shown in grey. Error bars show the variation in growth among three independent replicate cultures.

Finally, we observed a difference in growth characteristics that might result from ecological differentiation. Triplicate cultures were grown that showed that the two groups differ in heterotrophic growth characteristics in rich media developed for *Sulfolobus* in the laboratory [31]. The Red strains have a shorter lagging time, higher growth rate, and higher culture density than the Blue strains (Figure 5). The growth difference between the Red and Blue groups was statistically significant (*t* test, *p*<0.001). Although growth differences could result from many aspects of *Sulfolobus* physiology, these data provide a phenotypic basis for the definition of two species that is commonly suggested as necessary in microbial taxonomy [69],[70].

### Conclusions

The sequencing of multiple genomes from closely related strains of *S. islandicus* coexisting in the same hot spring has allowed us to identify evidence for sympatric speciation in this natural archaeal population. Without typical barriers to recombination associated with genetic distance, the mechanism of speciation is likely to be ecological differentiation among many loci throughout highly differentiated regions of the chromosome or by differential islands of gene content. Two incipient species are persistent in the Mutnovsky Volcano region, as MLSA of 42 strains collected from six springs in 2010 shows a very similar structure to that observed in 2000 (Figure 1A). This supports ecological differentiation that prevents competitive exclusion resulting in extinction of one type on this time scale [71],[72]. Speciation driven by ecological divergence rather than physical barriers to gene flow has been increasingly observed in sexual eukaryotes [11],[73]–[76]. Furthermore, the genomic pattern of differentiation under these circumstances has been theoretically predicted [77] and empirically demonstrated to exist in “continents” of differentiation using genomic analyses [65]. These initial reports identifying the genomic pattern of differentiation among species in model organisms of the domains *Archaea* and *Eukarya* point towards a possible unified genomic process of speciation across these two domains.

## Material and Methods

### Strain Isolation and DNA Extraction

Ninety-seven *S. islandicus* strains were isolated from eight hot springs located in the Mutnovsky Volcano region in the Kamchatka Peninsula (Russia), with specifications listed in Table S1. Cultures were colony isolated on four different media: DT (dextrin and tryptone) spread plate as described in [31],[78], DTO plate containing DT plate plus an overlay of 0.002% Gelrite (Sigma), DTS spread plate containing DT plate plus an overlay of 0.002% colloidal sulfur, and a DTSO plate containing a DTS plate as described above plus an overlay of 0.002% Gelrite (Sigma) as shown in Table S1. Each isolate was subjected to three additional rounds of colony purification, and then grown in liquid phase followed by DNA extractions as detailed elsewhere [30],[31].

### Multilocus Sequence Analysis (MLSA)

The set of seven MLSA loci including the primer sequence, PCR conditions, reactant concentrations, and sequencing conditions were selected as a subset of the 12 loci described in detail elsewhere [32]. Sequences of unique alleles are deposited in GenBank under the accession numbers HQ123504-HQ123512, HQ123518-HQ123527, HQ123532-HQ123534, and HQ123541-HQ123543, and JQ339286–JQ339304. MLSA data were evaluated for all seven loci using the Clonal Frame V1.2 software [35]. Runs of 250,000 iterations, after 100,000 burn-in iterations, were found to have reached convergence based on between run comparisons. Arlequin 3.5 [34] was used for performing F_ST_ calculations to test for differentiation using MLSA data between springs or between two years of sampling. Significance of F_ST_ values was determined through comparison to a permutation test, with *p*<0.05 considered significant.

### Genome Sequencing and Assembly

Ten strains from a single hot spring designated M.16, which is approximately 25 cm in diameter, were selected for *de novo* sequencing. Genomic preparations were done using a scaled up version of the protocol used for previous DNA extractions [30]. Briefly, 1,000 ml of *S. islandicus* cultures were concentrated and the pellet treated with 10 ml of 1× GES and 7.5 ml of 7.5 M ammonium acetate. After complete cell lysis, 15 ml of phenol∶chlorophorm∶iso-amyl alcohol (25∶24∶1) was added to the mix, homogenized, and subsequently centrifuged. Aqueous layer containing DNA and RNA mix was recovered for subsequent isopropyl alcohol and ethyl alcohol DNA purification. Extracted DNA was treated with 1 unit of RNAse I (New England Laboratories) for 30 min at 37°C. DNA quality and concentration was evaluated by gel electrophoresis and spectrophotometry. Genome sequencing was done using a 454 Life Sciences GS-FLX sequencer (Roche) at the University of Illinois Core Sequencing Facility (www.biotec.illinois.edu). All 10 strains were initially “shotgun sequenced” reaching 8–20× coverage. Six strains (M.16.02, M.16.22, M.16.23, M.16.40, M.16.43, and M.16.47) were further selected for a second round using “454 paired-end sequencing,” increasing the genome coverage to 28–51× (Table S5).

Genome assembly was done in three steps. First, reads were assembled using the GS De Novo Assembler V.2.0.00 (Roche). Second, the GS assemblies were evaluated using the Consed V.19 software [79], where contigs exceeding more than twice the expected average coverage were reassembled using the “miniassembly” tool with default settings. For the remaining contigs, a fragment of 700 bp was removed from both ends and the removed fragments reassembled individually with the miniassembly tool; if the miniassembly tool produced a single contig, then the fragment was joined back into the contig, but if more than one contig was formed from reassembly, then the fragments were left as new contigs to be resolved in the next assembly step. This second assembly step addresses two possible artifacts: (a) separating reads with sections of similar sequence, but belonging to different copies of repetitive or duplicated elements, forming short contigs with abnormally high coverage, and (b) resolving partially assembled reads with masked regions (not contributing to contig consensus) at both ends of a contig. The third assembly step used comparative genomics to organize and scaffold contigs of the draft genomes using the two completed M.16 genomes [30] followed by PCR sequencing of small gaps. MUMmer 3.0 [80], the “move contigs” tool from the Mauve V.2.3.1 [81] and ABACAS [82] software, was used to generate the draft genomes of the 10 newly sequenced strains described in Table 1. Comparative genomics and paired-end data predicted very few gaps in the draft genomes, none of which is likely longer than 4 or 7 Kb. These gaps were joined by short strings of “N” to artificially close draft genomes to facilitate gene prediction and genome analysis. Four of seven genomes from the Blue group and all three genomes from the Red group were closed to exclude possibility of missing genes in gaps of draft sequences that could contribute to ecological differentiation between them. The draft versions of all genomes were deposited as a Whole Genome Shotgun project at DDBJ/EMBL/GenBank under the accession AHJK00000000, AHJL00000000, AHJM00000000, AHJN00000000, AHJO00000000, AHJP00000000, AHJQ00000000, AHJR00000000, AHJS00000000, AHJT00000000. The version described in this paper is the first version, AHJK01000000, AHJL01000000, AHJM01000000, AHJN01000000, AHJO01000000, AHJP01000000, AHJQ01000000, AHJR01000000, AHJS01000000, AHJT01000000 and are also available at http://www.life.illinois.edu/Sulfolobus_islandicus. ORFs in the newly sequenced genomes were predicted and automatically annotated using the Rapid Annotation with Subsystems Technology (RAST) V2.0 software and FIGfams set of protein families [83].

Identification of the core genomic regions was done using the ProgressiveMauve algorithm [81] on the set of 12 M.16 genomes. The alignment contains regions shared among all genomes, shared by a subset, or unique to one genome. The blocks shared by all genomes were split where gaps of more than 20 alignment positions were found, resulting in 155 core region fragments. The cumulative length of the core region for each genome was recorded and the fraction that they represent of each strain genome is detailed in Table 1.

The core region was then analyzed using ClonalFrame with default parameters [35]. Runs of 10,000 iterations were found to have reached convergence based on between-runs comparisons. The clonal genealogy reconstructed by ClonalFrame is shown in Figure 1B.

### Homologous Recombination Analysis with ClonalOrigin

ClonalOrigin is a Bayesian method to perform approximate inference under the coalescent model with gene conversion [84] using whole microbial genomes [36]. Assuming the correctness of the clonal genealogy reconstructed by ClonalFrame (Figure 1B), ClonalOrigin reconstructs recombination events that represent a deviation from such vertical inheritance. Each recombination event is characterized by the genomic segment it affects, as well as an origin and destination on the clonal genealogy. By summarizing these last two properties across all events, we reconstructed the overall pattern of genetic flux between branches of the tree (Figures 2 and S1). We also studied the distribution of recombination segments along the genome and found no correlation with the level of polymorphism in 1, 000 bp windows of the alignment (Figure S2). The absolute recombination rate was estimated by ClonalOrigin to be 2.41×10^−4^ per site per coalescent unit of time. A coalescent unit of time is equal to the average length of a generation multiplied by the effective population size. ClonalOrigin also estimated the ratio of frequencies of recombination and mutation (ρ/θ) and the average tract length of recombination events (δ). The relative effect of recombination and mutation (r/m) was estimated using the formula of Jolley et al. 2005 [37]: r/m = (ρ/θ)*δ*π, where π is the average pairwise distance between two genomes. This formula is correct assuming that any two genomes are equally likely to recombine, but here we found (Figure 2) that recombination happens more often between genomes from the same lineage so that r/m might be overestimated by this formula. We also calculated r/m taking the formula above with π equal to the average pairwise distance of two genomes in the same lineage. This would be correct if recombination happened only between members of the same lineage, but here we know that recombination also happens across lineage boundaries (Figure 2). Thus, this second calculation is likely to underestimate r/m, and taken together, the two calculations above provide us with a lower and upper bound on r/m.

Recombination events were also evaluated by manual pairwise scanning methods using the Recombination Detection Program 3 software [85] for all core genomic regions. A genomic section was identified as evidence of a HR event after finding significant results (*p*<0.01) with at least three of the following four tests as implemented in the Recombination Detection Program 3 software: RDP, GENECONV, MaxChi, and Bootscan. Overall, these results show similar patterns to the ClonalOrigin analysis.

### Evaluation of Integrated Elements and Distribution of Variable Genome Components

Boundaries of variable segments from the Mauve alignment are defined where there are core regions greater than 5 kb. Variable gene segments that are smaller than 5 kb in length or contain less than half variable gene segments were excluded from analysis because the majority result from insertion elements.

Each variable segment was investigated for its distribution among the 12 genomes. This allowed us to identify composite segments with different distributions among strains. There was one segment per genome that was excluded from analysis due to a complicated pattern of shared and unique content that made segment boundaries very difficult to assign. In M.16.27, this segment is 74 kb long and located at 997,394–1,071,175 in the genome. To identify integrated mobile elements, variable gene segments were compared to a database of *Sulfolobus* mobile elements [40] including elements that are integrated into *S. islandicus* genomes [30],[86] using BLASTN (e<0.001, −F f) and to the NCBI nr database using BLASTX (e<1E-5, −F f).

BLASTN was used to compare variable segments to the rest of the *S. islandicus* genomes from the M.16 hot spring in order to assess Mauve's assignment of variation.

The genome in which each variable segment was longest was compared to each other genome in which the segment is present, and the level of nucleotide identity was calculated between each of them as the number of matching nucleotides divided by the length of the match as reported by BLASTN and averaged over all of the pairs. The coverage of the longest segment was also calculated pairwise as with percent identity, with the total length of matching nucleotides divided by the length of the longest segment and averaged over all pairs. Genes present in the variable segments that separate Blue from Red were compared to NCBI's nr database with BLASTP (e<0.001, −F f) if they were complete in every genome from either Blue or Red and if they were not core genes.

### Genomic Patterns of Differentiation

A table containing the position and nucleotide of every SNP in the core genome alignment was exported from the genome alignment using the “Export SNPs” tool from the Mauve software. SNPs found within and between the Blue and Red species were used for sliding window F_ST_ evaluations. F_ST_ values were calculated for sliding windows of 10,000 bps moving in 5,000 bp steps across the genome of M.16.27. Arlequin 3.5 [34] was used for calculating F_ST_ to test for differentiation in the M.16 populations. Low regions were defined as beginning and ending where windows of F_ST_ values were less than 0.5. F_ST_ values were not calculated for empty windows or where sequence found in M.16.17 is not present in all strains. F_ST_ values for genes were calculated using the same methods but applying the coordinates of each gene to SNPs exported from the core alignment from Mauve rather than sliding windows.

Pairwise d_N_/d_S_ ratios were calculated using the ORF clusters identified by MCL analysis [87]. All clusters containing a single copy of an ORF per genome (2,187) were evaluated for all pairwise d_N_, d_S_, and d_N_/d_S_ ratios using the SNAP program (http://hiv-web.lanl.gov/) [88] with the Nei and Gojobori method as described elsewhere [89]. Sequence alignments for clusters that resulted in d_N_/d_S_ values greater than 1.0 were manually checked to resolve homopolymer indels from the 454 sequence data.

### Growth under Heterotrophic Conditions

The 12 M.16 strains were evaluated for their growth characteristics in rich liquid media. As initially described by Whitaker et al. (2003) [31], culturing conditions were at pH 3.5, 75–78°C and with media containing a basal mineral solution, 0.1% Dextrin (Fluka) and 0.1% Tryptone (Difco). All cultures were initiated using exponentially growing cultures to inoculate 50 ml liquid cultures in 250 ml flasks at an estimated starting concentration of 2×10^3^ cells/ml. Cultures were incubated under static conditions and growth was followed by OD600 reads up to the sixth day of incubation.

## Supporting Information

Figure S1 Heatmap showing absolute number of recombinant events between donor/recipient pair of branches of the core genome phylogeny of 12 *S. islandicus* strains. For each pair observed, number of events (top) is compared to the number of expected events under the prior used in the ClonalOrigin model (bottom). As in Figure 2, recombination frequency is measured relative to its expectation under the prior used in the ClonalOrigin model and color coded according to the upper left color/magnitude legend (light blue and blue for rates below a 1∶1 ratio and yellow to red for rates above 1∶1). Light gray cells represent non-significant ratios with less than four observed and expected events. White shows number of events that match the prior expectations. Names of strains are color coded as Blue and Red groups.
(EPS)

Figure S2 Relationship between genetic distances and recombination frequency as measured using ClonalOrigin (A and B) or experimental data (C) over the range of genetic distances observed in *S. islandicus*. Recombination frequency is normalized to 1 being the maximum number of events observed. (A) *Sulfolobus islandicus* and (B) *Bacillus cereus* [1]; (C) blue circles, *Saccharomyces cerevisiae* [2]; red diamonds, *Streptococcus pneumoniae* [3]; green triangles, *Bacillus subtilis* [4]; purple squares, *Escherichia coli* [5].
(EPS)

Figure S3 Maximum likelihood phylogeny using the nucleotide sequences of S-layer protein from published *S. islandicus* strains [6]–[9]. Phylogeny was produced in MEGA 5 [10] using the GTR+G model determined to best fit the data using Modeltest [11]. Each node on this phylogeny has greater than 80% support out of 1,000 bootstrap replicates. Strains from this study are highlighted according to their species affiliation (Red or Blue) as in Figure 2.
(EPS)

Table S1 List of *S. islandicus* strains.
(XLSX)

Table S2 MLSA allele assignment for *S. islandicus* strains.
(XLSX)

Table S3 Pairwise F_ST_ values for each hot spring sample calculated from seven concatenated MLSA loci.
(XLSX)

Table S4 Variable genome segments among 12 *S. islandicus* genomes.
(XLSX)

Table S5 Sequencing statics for 10 new *S. islandicus* genomes.
(XLSX)

Text S1 Supporting references.
(DOCX)

## Abbreviations

HR: homologous recombination
MLSA: multilocus sequence analysis
RAST: Rapid Annotation with Subsystems Technology
SNPs: single nucleotide polymorphisms
