# Supplementary material for: Patterns of Gene Flow Define Species of Thermophilic Archaea
Source: PLoS Biol. 2012 Feb 21;10(2):e1001265. doi: 10.1371/journal.pbio.1001265 (PMC3283564; doi:10.1371/journal.pbio.1001265)
Supplement: Text S1 — Supporting references. (DOCX) [file pbio.1001265.s009.docx]

Supplemental References:

1. Didelot X, Lawson D, Darling A, Falush D (2010) Inference of homologous recombination in *Bacteria* using whole genome sequences. Genetics: genetics.110.120121.

2. Datta A, Hendrix M, Lipsitch M, Jinks-Robertson S (1997) Dual roles for DNA sequence identity and the mismatch repair system in the regulation of mitotic crossing-over in yeast. Proceedings of the National Academy of Sciences 94: 9757-9762.

3. Majewski J, Zawadzki P, Pickerill P, Cohan FM, Dowson CG (2000) Barriers to Genetic Exchange between Bacterial Species: Streptococcus pneumoniae Transformation. J Bacteriol 182: 1016-1023.

4. Zawadzki P, Roberts MS, Cohan FM (1995) The Log-Linear Relationship Between Sexual Isolation and Sequence Divergence in Bacillus Transformation is Robust. Genetics 140: 917-932.

5. Vulic M, Dionisio F, Taddei F, Radman M (1997) Molecular keys to speciation: DNA polymorphism and the control of genetic exchange in enterobacteria. Proceedings of the National Academy of Sciences 94: 9763-9767.

6. She Q, Singh RK, Confalonieri F, Zivanovic Y, Allard G, et al. (2001) The complete genome of the crenarchaeon Sulfolobus solfataricus P2. Proceedings of the National Academy of Sciences of the United States of America 98: 7835-7840.

7. Guo L, Brugger K, Liu C, Shah SA, Zheng H, et al. (2011) Genome Analyses of Icelandic Strains of Sulfolobus islandicus, Model Organisms for Genetic and Virus-Host Interaction Studies. J Bacteriol 193: 1672-1680.

8. Chen L, Brugger K, Skovgaard M, Redder P, She Q, et al. (2005) The Genome of Sulfolobus acidocaldarius, a Model Organism of the Crenarchaea. Journal of Bacteriology 187: 4992-4999.

9. Reno ML, Held NL, Fields CJ, Burke PV, Whitaker RJ (2009) Biogeography of the *Sulfolobus islandicus* pan-genome. Proceedings of the National Academy of Sciences 106: 8605-8610.

10. Tamura K, Peterson D, Peterson N, Stecher G, Nei M, et al. (2011) MEGA5: Molecular Evolutionary Genetics Analysis using Maximum Likelihood, Evolutionary Distance, and Maximum Parsimony Methods. Molecular Biology and Evolution.

11. Posada D, Crandall KA (1998) MODELTEST: testing the model of DNA substitution. Bioinformatics 14: 817-818.

12. Held, N. L., Herrera, A., Cadillo-Quiroz, H. and Whitaker, R. J. (2010) CRISPR associated diversity within a natural population of *Sulfolobus islandicus*. PLoS ONE 5: e12988.
